# Functional Improvement of Human Cardiotrophin 1 Produced in Tobacco Chloroplasts by Co-Expression with Plastid Thioredoxin m

**DOI:** 10.3390/plants9020183

**Published:** 2020-02-02

**Authors:** María Ancín, Ruth Sanz-Barrio, Eva Santamaría, Alicia Fernández-San Millán, Luis Larraya, Jon Veramendi, Inmaculada Farran

**Affiliations:** 1 Institute for Multidisciplinary Research in Applied Biology, UPNA, 31006 Pamplona, Spain; maria.ancin@unavarra.es (M.A.); alicia.fernandez@unavarra.es (A.F.-S.M.); luis.larraya@unavarra.es (L.L.); jon@unavarra.es (J.V.); 2 National Centre for Biotechnology, Plant Molecular Genetics Department, CSIC, 28049 Madrid, Spain; 3 Hepatology Program, University of Navarra, CIMA, E-31008 Pamplona, Spain; evasmaria@unav.es; 4 CIBERehd, Instituto de Salud Carlos III, 28220 Majadahonda, Madrid, Spain

**Keywords:** cardiotrophin-1, thioredoxin, plastid transformation, bioactivity, tobacco

## Abstract

Human cardiotrophin 1 (CT1), a cytokine with excellent therapeutic potential, was previously expressed in tobacco chloroplasts. However, the growth conditions required to reach the highest expression levels resulted in an impairment of its bioactivity. In the present study, we have examined new strategies to modulate the expression of this recombinant protein in chloroplasts so as to enhance its production and bioactivity. In particular, we assessed the effect of both the fusion and co-expression of Trx m with CT1 on the production of a functional CT1 by using plastid transformation. Our data revealed that the Trx m fusion strategy was useful to increase the expression levels of CT1 inside the chloroplasts, although CT1 bioactivity was significantly impaired, and this was likely due to steric hindrance between both proteins. By contrast, the expression of functional CT1 was increased when co-expressed with Trx m, because we demonstrated that recombinant CT1 was functionally active during an in vitro signaling assay. While Trx m/CT1 co-expression did not increase the amount of CT1 in young leaves, our results revealed an increase in CT1 protein stability as the leaves aged in this genotype, which also improved the recombinant protein’s overall production. This strategy might be useful to produce other functional biopharmaceuticals in chloroplasts.

## 1. Introduction

Proteins are widely used in medicine as diagnostic reagents, vaccines and drugs, and this creates a strong demand for the production of recombinant proteins on an industrial scale. Commercial protein production has traditionally relied on microbial fermentation and mammalian cell lines. However, the use of plants as bioreactors, a technology known as plant molecular farming, offers several advantages over traditional systems such as reduced manufacturing costs, minimized risk of contamination with human pathogens or toxins, and production that is easily scalable [1]. Therefore, transgenic plants are potentially one of the most economical systems for large-scale production of recombinant proteins for industrial and pharmaceutical uses [2].

Among the approaches for generating transgenic plants, chloroplast transformation is showing promise as an expression system [3,4], mainly due to the chloroplast’s enormous capacity to accumulate foreign proteins, reaching levels up to 70% of the leaf total soluble protein [5,6]. Several additional advantages to chloroplast technology can be noted, particularly the maternal inheritance of plastids and their DNA [7], which minimizes outcrossing of transgenic pollen with related weeds or crops, and thus increases the biosafety of genetically modified plants. Furthermore, the presence of chaperones and enzymes in the chloroplast facilitates the assembly and correct folding of multiple proteins with appropriate disulfide bonds [8]. Other advantages are the possibility to express multiple genes organized in operons [9] and the elimination of pleiotropic effects and gene silencing [10]. In contrast, the main problem of plant molecular farming is the high cost of purifying the recombinant proteins. However, oral delivery of chloroplast-derived recombinant proteins could cut down the costs of protein purification, storage and transportation [11]. These aspects make the plastid genome of higher plants an attractive target for engineering. 

Many proteins with pharmaceutical interest have been produced in transplastomic plants in recent years [3]. Among them, the production of human cardiotrophin-1 (CT1), a member of the interleukin-6 family of cytokines, can be highlighted due to its extremely high therapeutic potential. This protein was first identified by its ability to induce the hypertrophic response in cardiac myocytes [12], but subsequent studies have shown the protective role of CT1 in different organs [13]. The first and only attempt to produce human CT1 in plants was accomplished by Farran and co-workers [14], obtaining a high expression level in tobacco chloroplasts. However, the highest CT1 levels were obtained under continuous light where the bioactivity of the recombinant protein was impaired. Therefore, expression of a fully functional CT1 could be improved following other strategies, whereby thioredoxins (Trxs) could acquire an important role. 

Trxs are ubiquitous, small, heat-stable, and soluble proteins with a compact globular structure and high translatability, and they catalyze oxidoreductase reactions by a thiol-disulfide exchange mechanism [15]. In this way, reduced Trxs interact with specific disulfide sites to increase or decrease the activity of target proteins. Additionally, Trxs have been shown to promote the folding of proteins in a redox-independent manner, either by enhancing the refolding activity of other molecular chaperones or by directly promoting protein folding [16]. Indeed, chaperone-like properties have been attributed to Trxs over the years [17,18,19]. In microbial systems, TrxA from *Escherichia coli* has become a widely used biotechnological tool, as a separate co-produced protein or as a fusion tag, to improve the expression, solubility and folding of heterologous proteins [20,21,22,23,24]. The fusion strategy generally results in reliably high protein yields and can simplify protein purification by affinity chromatography. However, it also leads to considerations about how the fusion partner may affect folding or activity, and introduces an additional problem into the downstream processing because site-specific cleavage is needed [25].

In parallel to bacterial expression systems, the endosymbiotic origin of plant plastids means that Trx technology might also be applicable to chloroplast expression platforms for plant-produced proteins. However, unlike bacteria and animals, plants have an extended Trx system composed of about 20 major classes including classical Trxs and Trx-like proteins, localized in different sub-cellular compartments [26]. In chloroplasts, five classical Trx isoforms have been reported: f, m, x, y, and z [27,28]. Among them, Trx m can be found in oxygenic prokaryotes, algae and terrestrial plants, and it shows a high similarity to heterotrophic anoxygenic Trxs [29]. The structural analysis of Trx m [30,31] has indicated that both the three-dimensional conformation and the surface surrounding the active site are structurally and functionally very similar to the TrxA from *E. coli* [32]. In this sense, Trx m might be a good candidate to modulate heterologous protein expression in plant chloroplasts. In fact, plastid Trxs have already been used as solubility and stability enhancers of recombinant proteins in the tobacco chloroplast. Both fusion and co-expression of the tobacco plastid Trxs f and m with human serum albumin (HSA) have been reported [33]. The Trx fusion strategy increased the expression of HSA in chloroplasts 3–4 fold, mainly due to the high stability of the fused Trx-HSA proteins, but failed to prevent the formation of protein bodies within chloroplasts. However, a direct relationship between solubilization of HSA aggregates and Trx f or m overexpression in tobacco plants co-expressing both proteins from the chloroplast genome was observed. With this background, in this study we have explored the use of plant Trx m as an enhancer element for the production of functional human CT1 in tobacco chloroplasts by using both fusion and co-expression strategies. Our results demonstrate that the co-expression of Trx m and CT1 from the chloroplast genome increases CT1 stability, but also its bioactivity inside the chloroplast, leading to the production of a fully functional CT1, while improving overall recombinant protein production in tobacco plants. This work constitutes the first evidence that Trxs could exert an important role in modulating the bioactivity of recombinant proteins in plant chloroplasts.

## 2. Results

### 2.1. Generation of Transplastomic Tobacco Plants Expressing Human CT1 Fused or Co-Expressed with Trx m

**Figure 1:**
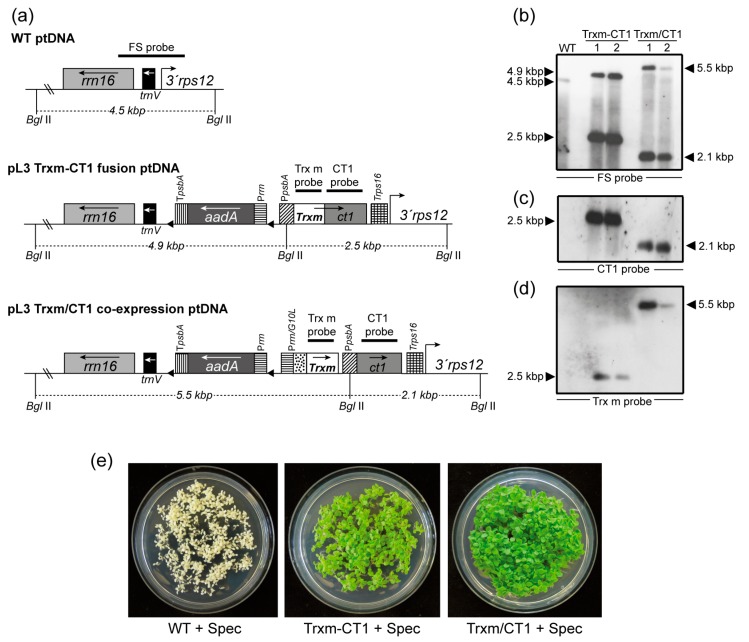
Integration of *Trxm* and *ct1* genes by fusion or co-expression into the plastid genome and homoplasmy verification. (**a**) Map of the WT, Trxm-CT1 fusion and Trxm/CT1 co-expression plastid DNA. Transgenes were cloned into the intergenic region between the *rrn16/trnV* and *3′rps12* genes. The Trxm-CT1 fusion is driven by the *psbA* promoter and the *rps16* terminator. In co-expression, CT1 is also driven by P*psbA* but Trx m is driven by the *rrnG10L* promoter. Arrows within boxes show the direction of transcription. The selectable gene *aadA* is driven by the *rrn* promoter and *psbA* terminator. Probes for the Southern blot are shown over the corresponding sequences. The sizes of the predicted bands when DNA was digested with *Bgl*II are indicated. (**b**–**d**) Southern blot analysis for WT and two independent plants per construct is shown. The same blot was probed with FS (**b**), CT1 (**c**) and Trx m (**d**) probes. (**e**) Seed assays confirming homoplasmy of regenerated plants. WT seedlings bleach out on the spectinomycin-containing medium whereas seedlings from both transplastomic genotypes are resistant (green color). *rrn16, trnV*, *3′rps 12*: original sequences of the chloroplast genome; *aadA*: aminoglycoside 3′-adenylyltransferase; P*rrn*: 16SrRNA promoter; P*psbA*: *psbA* promoter and 5′-untranslated region; T*psbA*: *psbA* terminator; P*rrnG10L*: *rrn* promoter and the gene 10 leader from phage T7; T*rps16*: *rps16* terminator; *Trxm*: thioredoxin m; *ct1*: cardiotrophin-1; Trxm-CT1: fusion plants; Trxm/CT1: co-expression plants.

To analyze whether plastidial Trx m could modulate the expression of recombinant CT1 in chloroplasts, both fusion and co-expression strategies were examined. For the fusion construct, a Trxm sequence corresponding to the mature peptide was translationally fused to the ct1 sequence (Figure 1a). In the middle of both sequences, the flexible hinge tetrapeptide GPGP was included in order to reduce steric hindrance between both proteins and facilitate protein fusion assembly [34]. The fusion gene was expressed from the tobacco psbA promoter and 5′-UTR regulatory sequences, which allowed very high levels of recombinant proteins to be expressed in chloroplasts [33,34]. The construct was introduced into the chloroplast transformation pL3 vector, which integrates transgenes between the rrn16/trnV and 3′rps12 genes in the inverted repeat region of the chloroplast genome (Figure 1a). This vector also includes the aadA gene from E. coli, which confers resistance to both spectinomycin and streptomycin and is driven by the constitutive promoter of the 16S rRNA operon (rrn) and the psbA terminator. For the co-expression vector, the Trxm sequence was expressed from the constitutive tobacco rrn promoter followed by the T7 phage gene 10 leader sequence (PrrnG10L), which is one of the strongest known expression signals in plastids [35]. The PrrnG10LTrxm cassette was introduced into the pL3-PpsbA-CT1 vector [14] upstream of the ct1 gene (Figure 1a), which was driven by the tobacco psbA promoter and 5′-UTR. Therefore, both the ct1 and Trxm genes were expressed using different promoters to avoid possible deletions via homologous recombination [36], but shared a common terminator (Trps16, Figure 1a). 

Chloroplast transformation was achieved through bombardment of gold microprojectiles coated with the previously described vectors into in vitro-grown Petite Havana tobacco leaves as previously described [37]. The leaves were placed in a regeneration medium that included spectinomycin as a selection agent. Shoots regenerated from these leaves were subjected to several rounds of selection under the same conditions in order to obtain homoplasmic plants for both fusion and co-expression transformed genotypes (hereafter referred to as Trxm-CT1 and Trxm/CT1, respectively). Successful transformation of the plastid genome and correct site-specific integration of the transgenes by homologous recombination between the *rrn16*/*trnV* and 3′*rps12* genes were assessed by Southern blot analysis. The DNA from regenerated and untransformed (WT) plants was digested with the *Bgl*II restriction enzyme, which cuts two positions flanking the insertion site outside the left and right recombination regions and one position in the *psbA* promoter (Figure 1a). The FS probe, which is homologous to the flanking regions *rrn16*/*trnV* and 3′*rps12*, identified a 4.5-kbp fragment in the WT plant, 4.9- and 2.5-kbp fragments in plants expressing the Trxm-CT1 fusion, and 5.5- and 2.1-kbp fragments in plants co-expressing Trx m and CT1 (Figure 1b). Two independently transplastomic lines per construct are shown. The absence of a 4.5-kbp fragment in transgenic genotypes indicates homoplasmy (Figure 1b). However, line 2 of the Trxm/CT1 genotype showed a slight 4.5-kbp fragment that would indicate heteroplasmy. Therefore, this line was discarded and line 1 was used to carry out the following experiments. When the blot was stripped and reprobed with gene-specific probes, the expected hybridization pattern was observed in all the transplastomic genotypes for both CT1 (Figure 1c) and Trx m probes (Figure 1d). As expected, CT1-specific hybridization was absent in the WT plants (Figure 1c), but Trx m-specific hybridization was also not visible. Although *Trxm* is an endogenous tobacco gene, the nuclear DNA levels were likely below the detection limit of the labeling system used. To ultimately confirm homoplasmy, seeds from the T_0_ generation were germinated in vitro on a spectinomycin-selective medium. A lack of segregation for spectinomycin resistance in the T_1_ generation demonstrated homoplasmy (Figure 1e). 

### 2.2. Expression Levels of Human CT1 Fused or Co-Expressed with Trx m in Tobacco Chloroplasts

**Figure 2:**
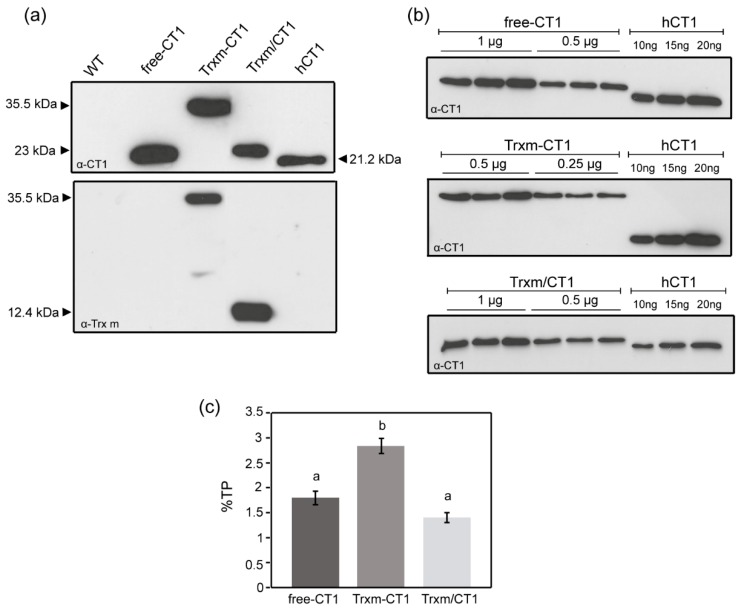
CT1 accumulation in fusion and co-expression tobacco plants. (**a**) Western blot analysis of CT1 and Trx m. Ten micrograms of total protein (TP) extracted from WT and transplastomic genotypes were loaded onto a 13% SDS-PAGE gel. Commercial human CT1 (20 ng) was used as standard. (**b**) Quantification of recombinant CT1 in transplastomic genotypes by densitometry of Western blots. A dilution series of commercial hCT1 was included. Protein loading, whose amounts (µg of total protein) are indicated over the blots, was adjusted to become comparable to the hCT1 standard. Biological replicate extracts from three different plants of each genotype are shown. (**c**) Representation of CT1 quantification in young tobacco leaves. Results are the mean ± SE of two measurements for three independent transgenic plants per construct and are shown as a percentage of TP. Different letters indicate significant differences (t-test, *p* ≤ 0.01). Free-CT1: free-CT1 expressing plants; Trxm-CT1: fusion plants; Trxm/CT1: co-expression plants; hCT1: commercial human cardiotrophin 1.

To determine the accumulation of CT1 protein in chloroplasts, plants from the T_1_ generation of homoplasmic fusion and co-expression genotypes were analyzed using the previously generated CT1 expressing plants as control [14] (hereinafter referred as free-CT1). Total protein (TP) from young leaves was extracted and analyzed by Western blot using a specific CT1 antibody (Figure 2a, upper blot). For all genotypes, the antibody recognized a specific protein of the expected molecular mass, but no immunoreactive polypeptide was present in the WT plant extracts, indicating that the detected signal was specific for CT1. Monomers of CT1 were detected at approximately 23 kDa in free-CT1 and Trxm/CT1 co-expressing genotypes, which were slightly larger than the size of commercial human CT1 (21.2 kDa) as a result of the polyhistidine tag. Likewise, monomers corresponding to the Trxm-CT1 fusion protein (35.5 kDa) were also detected. The extracted protein was also analyzed by Western blot with specific Trx m antibody (Figure 2a, lower blot). Monomers of both Trx m (12.4 kDa) and Trxm-CT1 (35.5 kDa) were detected in the co-expressing and fusion genotypes, respectively. In this blot, endogenous Trx m was undetectable in WT plants due to its low expression level compared to transplastomic genotypes.

In order to analyze putative differences in recombinant protein expression among genotypes, the accumulation of CT1 protein in young fully expanded leaves was quantified by the densitometry of Western blots. Serial dilutions of TP extracted from the different transplastomic plants and commercial human CT1 standard were loaded on the blot (Figure 2b), and the intensity of the immunoreacted bands was compared using Gene-Tools analyzer software. The transplastomic Trxm-CT1 fusion genotype accumulated recombinant CT1 at 2.8% of TP, which significantly increased (by up to 60%) the amount of CT1 produced by the free-CT1 genotype (Figure 2c). CT1 accumulated at 1.4% of TP when co-expressed with Trx m, showing no significant differences with the free-CT1 genotype expression level (1.8%) (Figure 2c).

**Figure 3:**
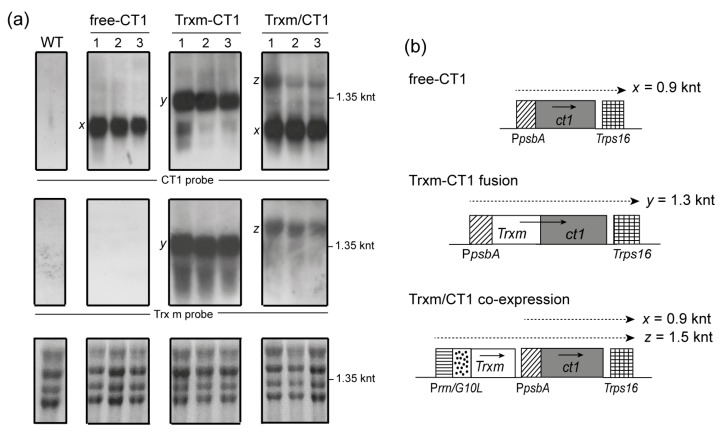
Levels of *ct1* and *Trxm* transcripts in transgenic tobacco plants. (**a**) A Northern blot analysis was performed with RNA extracted from tobacco leaves of WT and transplastomic genotypes. Three plants for each construct were analyzed. Twenty micrograms of total RNA were electrophoresed, blotted and hybridized with CT1 (top panels) and Trx m (middle panels) specific probes. Ethidium bromide stained rRNA was used to assess loading (lower panels). (**b**) Expected transcription patterns for the different constructs integrated into the chloroplast genome. Horizontal discontinuous arrows above genes show monocistronic (x and y) and dicistronic (z) transcripts, and their expected sizes are indicated. Arrows within boxes show the direction of transcription. Free-CT1: free-CT1 expressing plants; Trxm-CT1: fusion plants; Trxm/CT1: co-expression plants.

Transcript abundance was examined using Northern blot analysis to determine whether changes in ct1 mRNA accumulation could account for the observed changes in CT1 protein accumulation. Total RNA extracted from the same leaf samples as used for CT1 quantification was employed for the analysis. Abundant monocistronic transcripts of expected size were detected in all genotypes via hybridization with the CT1 probe (0.9 knt in free-CT1 and Trxm/CT1 genotypes; 1.3 knt in the Trxm-CT1 fusion genotype), without a great difference in the amount of transcripts between them (Figure 3a, upper panels: x and y). In addition to the monocistronic ct1 transcripts (0.9 knt, x) produced from the psbA promoter in the Trxm/CT1 co-expressing genotype, dicistronic transcripts (1.5 knt, z), which include the Trxm and ct1 sequences from the rrnG10L promoter, were also detected (Figure 3a and b). Overall, our data revealed a lack of a relationship between levels of monocistronic ct1 transcripts and CT1 protein accumulation in all the transgenic genotypes, which suggests that post-transcriptional factors could account for the different levels of protein accumulation.

The blot was stripped and hybridized against a Trx m-specific probe (Figure 3a, middle panels). As expected, no transcripts of the endogenous Trxm gene were detected in WT and free-CT1 tobacco genotypes as a consequence of its low expression level (under the detection limit of the labeling system). Trxm-CT1 fusion plants accumulated high levels of Trxm monocistronic transcripts (1.3 knt, y) and confirmed the hybridization pattern seen above for the CT1 probe. However, only Trxm/ct1 dicistronic transcripts (1.5 knt, z) were observed in Trxm/CT1 co-expressing plants, which is likely due to the absence of a terminator after the Trxm sequence (Figure 3b).

### 2.3. Stability of Human CT1 Expressed in Tobacco Chloroplasts

**Figure 4:**
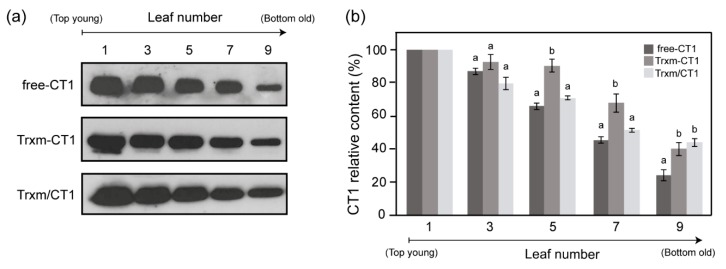
CT1 protein accumulation in tobacco chloroplasts as a function of leaf age. (**a**) Comparison of CT1 accumulation in a developmental series of five alternating leaf samples from the top to the bottom of the plant. Representative Western blots are shown. For each genotype, 2 µg of total protein were loaded. (**b**) CT1 densitometric quantification of tobacco leaves in different developmental stages. Leaves are numbered from the top (youngest) to the bottom (oldest) of the plant: 1, 3, 5, 7, and 9. Results are the mean ± SE of two measurements for three independent transgenic plants per construct. For each leaf, different letters indicate significant differences (t-test, *p* ≤ 0.05). Free-CT1: free-CT1 expressing plants; Trxm-CT1: fusion plants; Trxm/CT1: co-expression plants.

To examine the accumulation of recombinant CT1 in leaves at different stages of development, TP extracts from the different transgenic genotypes were prepared from leaves harvested at different positions along the plant and analyzed by Western blot (Figure 4a). In general, the largest amounts of CT1 were detected in young leaves (leaves 1 to 3 from top), whereas old leaves showed the smallest amounts. However, significant differences in the CT1 accumulation patterns among leaves were seen between transgenic genotypes. As the chloroplast capacity for protein biosynthesis declines with leaf age, the age-dependent decrease in foreign protein accumulation provides a good indicator of protein stability [5]. Thus, the amount of CT1 accumulated in old leaves relative to young leaves was quantified by densitometric analysis of Western blots and taken as a marker of CT1 stability within the chloroplast (Figure 4b). The proportion of old to young leaf CT1 (expressed as a percentage) in the free-CT1 plants was around 25%, while both Trx m fusion and co-expression genotypes reached 40%. These data reveal an increase in CT1 protein stability in old leaves of Trxm-CT1 and Trxm/CT1 plants, with Trx m most likely having an important role in this modulation.

### 2.4. Fusion and Co-Expression of Chloroplast-Expressed CT1 with Trx m Modulates the Protein’s Function

**Figure 5:**
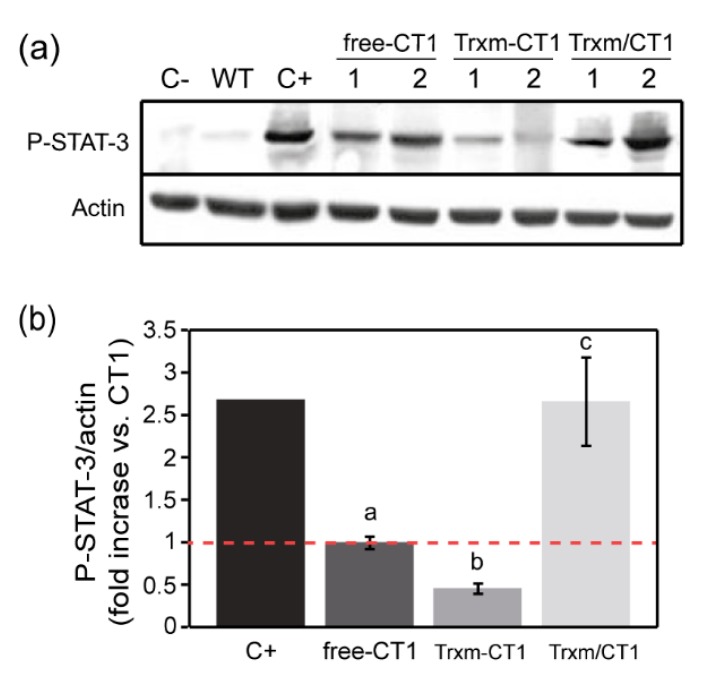
Bioactivity of recombinant human CT1 produced in tobacco chloroplasts. (**a**) Representative Western blot for P-STAT-3 (Tyr705) and actin (loading control) in the HepG2 cell line. Cells were stimulated with 25 ng per well of commercial hCT1 as positive control (C+). Cells treated with WT crude extract and non-stimulated cells (C-) were used as negative controls. For all the transplastomic genotypes, cells were treated with plant extract equivalent to 25 ng per well of soluble recombinant CT1. Two independent plants per transplastomic genotype are shown. (**b**) Densitometric analysis of P-STAT-3 in stimulated HepG2 cell line extracts. The results are presented as the fold increase relative to the free-CT1 genotype (= 1) using the ratios between the densities of the P-STAT-3 bands and the corresponding actin bands. The data are presented as the mean ± SE of three individual experiments (6 plants/genotype). Different letters indicate significant differences (t-test, *p* ≤ 0.05). P-STAT-3: phosphorylated-signal transducer and activator of transcription-3; free-CT1: free-CT1 expressing plants; Trxm-CT1: fusion plants; Trxm/CT1: co-expression plants.

To test the biological activity of CT1 fused and co-expressed with Trx m, the ability of CT1 to induce phosphorylation of the signal transducer and activator of transcription-3 (STAT-3) was measured in a human hepatocarcinoma cell line (HepG2). WT plant extracts showed no activity in this assay (Figure 5a), indicating that there is no endogenous plant compound with the ability to phosphorylate the STAT-3 protein (P-STAT-3). In contrast, all the extracts from the transplastomic genotypes induced such phosphorylation, although significant differences in the ability of recombinant CT1 to phosphorylate STAT-3 were seen among transgenic plants (Figure 5a). The pattern shown by actin band intensities indicates that the observed differences in P-STAT-3 levels were not the result of loading variability. The amount of P-STAT-3 protein was quantified by densitometric analysis. As reported before [14], the ability of free-CT1 to induce STAT-3 phosphorylation was significantly reduced (around 60 %) in comparison to commercial hCT1 (Figure 5b). The ability of the Trxm-CT1 fusion protein to activate kinases was equally reduced, showing a decrease of up to 80% in P-STAT-3 levels compared to the commercial hCT1 (Figure 5b). However, this alteration in CT1 functionality could be neutralized by co-expressing CT1 and Trx m in chloroplasts, where recombinant CT1 activity reached the levels of commercial hCT1 (Figure 5b). Thus, our results show a functional improvement in recombinant human CT1 produced in tobacco chloroplasts when co-expressed with Trx m. 

**Figure 6:**
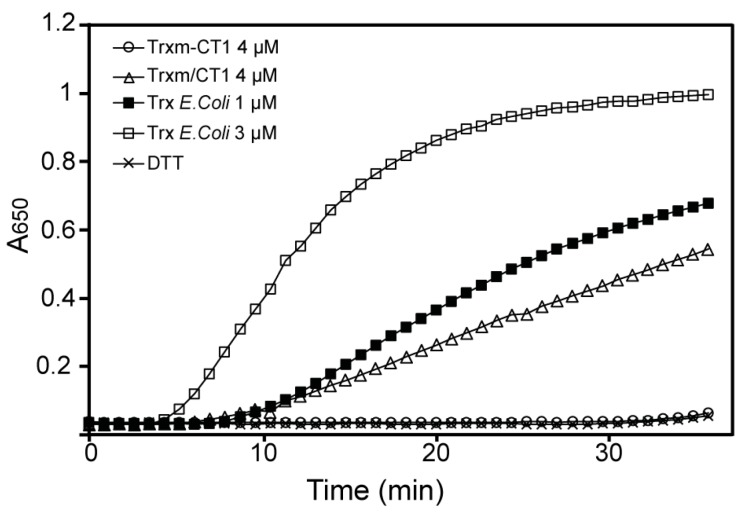
Disulfide oxidoreductase activity of Trx m protein from Trxm-CT1 and Trxm/CT1 genotypes. The dithiothreitol (DTT)-dependent insulin reduction assay of Trx m fused and co-expressed with CT1 was determined in a reaction mixture containing 4 μM of purified Trxm-CT1 fusion protein or 4 μM of purified Trx m/CT1 protein, supplemented with 0.5 mM DTT. Assays in the absence of Trx showing no activity were used as negative controls. Trx from *E. coli* was used at 1 and 3 μM as positive control.

To investigate whether the disulfide reductase activity of the Trxm-CT1 fusion protein was also altered, its ability to reduce the insulin β-chain was tested using the turbidimetric insulin precipitation assay [38]. For that purpose, the His-tagged Trx m proteins were purified by affinity chromatography from Trxm-CT1 or Trxm/CT1 tobacco leaf extracts using affinity columns packed with Ni-NTA agarose. Purity of the recombinant proteins was checked by SDS-PAGE followed by Coomassie staining. The Trxm-CT1 fusion protein failed to reduce insulin in the presence of dithiothreitol (DTT), while commercial E. coli Trx accelerated the insulin reduction in a concentration-dependent manner, as shown by the increase in turbidity at 650 nm (Figure 6). However, the oxidoreductase activity of Trx m protein purified from the co-expression Trxm/CT1 genotype seems to be preserved (Figure 6). Altogether, our results indicate that both oxidoreductase activity and STAT-3 phosphorylase induction were impaired in the Trxm-CT1 fusion protein produced in tobacco chloroplasts.

## 3. Discussion

CT1 is a cytokine with many ascribed functions in the cell and excellent therapeutic potential [13]. Hence, the demand for this biopharmaceutical is expected to increase and it would be wise to ensure its availability in significantly large amounts and in a cost-effective way. Among different plant production platforms, transgenic chloroplasts offer an enormous capacity to accumulate foreign proteins, mainly due to the high number of chloroplasts per cell and the high copy number of the plastid genome [39]. Human CT1 was previously expressed in tobacco chloroplasts at relatively high levels [14], although their functionality was impaired when plants were grown under high-light conditions, most likely due to improper protein folding. In plants, adequate targeting of foreign proteins to chloroplast sub-compartments seems to be of vital importance to achieve properly folded and functional polypeptides, suggesting that chaperones have a key role in these processes [40]. Previous reports have shown that the Trx protein fusion or co-expression strategies are useful for the correct folding of recombinant proteins in microbial systems and that the expression of functional proteins is increased by co-expression of molecular chaperones [41]. We have previously reported that the plastid Trxs f and m, which exhibit chaperone-like activity [19], can equally modulate the expression of recombinant HSA in transgenic chloroplasts of tobacco plants [33]. We have here investigated whether plastid Trx m (of prokaryotic origin) is a good heterologous partner for the expression of a fully functional CT1 in chloroplasts. For that purpose, we generated two different transplastomic tobacco genotypes where CT1 was expressed either fused or co-expressed with Trx m from the chloroplast genome. We focused on Trx m because no differences in protein modulation and chaperone-like properties between the f- and m-type Trxs had previously been observed [19,33].

Our results demonstrated an improvement in CT1 accumulation mediated by Trx m fusion, reaching levels of 2.8% TP in young fully developed leaves, whereas CT1 co-expressed with Trx m accumulated at a similar level to free-CT1 plants (1.4% and 1.8%, respectively; Figure 2b). Taking into account the ratio between soluble and total protein in the leaves, the amount of CT1 accumulated in the Trx m fusion genotype was about 5.1% of total soluble protein (TSP), which represents an increase of up to 60% compared to the 3.2% of TSP in the free-CT1 genotype (Figure 2b; [14]). A much greater increase in expression (3.5-fold) has been reported for Trx-HSA fusions in tobacco leaves, although these polypeptides accumulated in large protein bodies within the chloroplast [33]. The Trx m co-expression approach failed to increase CT1 accumulation within the chloroplasts of young leaves, as also happened for plastid Trx co-expression with HSA [33]. In bacterial expression systems, different behaviors of *E. coli* Trx-fusion and co-expression strategies regarding protein expression and solubility have also been reported [41,42]. The increase in CT1 protein accumulation observed in the fusion genotype here could not account for differences at the mRNA level because *ct1* monocistonic transcripts were detected in similar amounts in all the transplastomic plants (Figure 3). 

So, it can be postulated that other post-transcriptional factors must be influencing CT1 accumulation within chloroplasts in the Trxm-CT1 genotype, such as mRNA translatability or protein stability. Indeed, the efficiency of translation initiation seems to be an important feature for high-level expression of heterologous genes in plastids, where the sequence immediately downstream of the start codon has the main role [35]. Modifications or extensions of the N-terminus of the desired protein have often resulted in an increase in expression levels [43,44,45,46,47,48,49]. Thus, the high Trxm-CT1 protein accumulation in chloroplasts could be explained, at least in part, as being due to an improvement in translatability. Concerning the stability of CT1 protein in tobacco chloroplasts, our results indicated that the amount of recombinant protein declined as the leaves aged in the free-CT1 genotype (Figure 4; [14]). This age-dependent decline has previously been reported in chloroplast expression systems [49,50,51] and is a consequence of the high proteolytic activity of senescing leaves [52], which degrades both endogenous and foreign proteins. However, CT1 accumulation in older leaves increased when CT1 was fused or co-expressed with Trx m (Figure 4), which suggests that to some extent Trx m protects the CT1 protein from degradation inside the chloroplast. The well-established role of plastid Trxs in protein folding and degradation in the chloroplast [53], or its previously described chaperone activity [19,33], could account for the increased stability of CT1 in older leaves, at least for the Trxm/CT1 co-expression genotype. However, since the oxidoreductase activity of Trx m was impaired when fused at the N-terminal position of the CT1 sequence (Figure 6), the redox-independent chaperone properties of Trx m [19] could support the increased stability of the Trxm-CT1 fusion protein. In bacteria, the chaperone properties of TrxA have also been proposed to contribute to scFv folding and stability because a catalytic cysteine Trx mutant was still effective in supporting both folding and functionality of the recombinant protein [22]. Alternatively, Trx m could confer stability to the fused CT1 by protecting it from proteolytic degradation, as previously reported for chloroplast-expressed fusions of stable proteins, like GFP or GUS, with less stable partners [34,44,54,55]. This fact, together with translatability, could in turn explain the increased accumulation of Trxm-CT1 in young leaves. It is notable that the increased stability of CT1 observed in aged leaves of the Trxm/CT1 co-expression genotype (Figure 4), which resulted in a higher overall production of CT1 in Trxm/CT1 tobacco plants than in the free-CT1 genotype, contrasted with the two genotypes exhibiting similar CT1 expression levels in young leaves (Figure 2c). Nevertheless, we cannot rule out the role of Trx m in regulating transcriptional or translational activity in old leaves. Further experiments would be required to confirm this possibility.

Once achieved, the expression of recombinant human CT1 in tobacco chloroplasts and its ability to induce phosphorylation of STAT-3 in a human hepatocarcinoma cell line was measured (Figure 5). Our results showed that the CT1 produced in tobacco leaves was fully active when co-expressed with Trx m, whereas its functionality decreased in free-CT1 and Trxm-CT1 fusion genotypes. A reduction in CT1 bioactivity in plants grown under 32 h of continuous light has been reported [14], probably following the accumulation of harmful reactive oxygen species (ROS) in chloroplasts under these photo-oxidative stress conditions. In the present work, tobacco plants were grown under high intensity light in a greenhouse. Thus, Trx m could improve CT1 bioactivity in the co-expressing genotype by counteracting ROS production under those high light conditions. Peroxidases (Prxs) display important functions as antioxidants, regulating ROS concentrations in chloroplasts. The requirement for Trxs in the regeneration of thiol peroxidases and other antioxidant enzymes is well documented in vitro [56]. In particular, Trx m has been suggested to play an important role in the regeneration of enzymes involved in antioxidant mechanisms like Prxs and methionine sulfoxide reductases [57,58]. In addition, it has been reported that tobacco plants overexpressing Trx m are notably more tolerant (than Wt and Trx f-overexpressing plants) to oxidative stress conditions [59], providing in vivo evidence for the role of Trx m in antioxidant activity in chloroplasts. Hence, the co-expression of Trx m with CT1 in the chloroplast could have a protective effect on this protein. Alternatively, the increase in CT1 bioactivity observed in co-expressing plants could also be explained by the chaperone-like properties of Trxs [17,18,19,33]. Because the redox-independent foldase activity of Trx m has been demonstrated [19], the co-expressed Trx m could assist the correct folding and functionality of CT1 in the chloroplast. Finally, it cannot be ruled out that the co-expressed Trx m might trigger the redox-activation of another protein, which leads to an indirect effect on CT1 functionality. In fact, several chloroplast chaperones and co-chaperones have been suggested as potential targets for plastid Trxs [60,61]. 

The impaired functionality of Trxm-CT1 fusion protein produced in tobacco leaves may be due to incorrect protein folding caused by Trx m fusion at the amino terminus of CT1. Indeed, the disulfide reductase activity of the Trx m fused to CT1 was also damaged (Figure 6), as demonstrated by the insulin reduction assay [38]. Similarly, the foldase activity of Trx m in the fusion protein could also be affected. It is known that the presence of affinity tags may affect important characteristics or functions of the protein of interest [62] and may interfere with protein activity [63]. Alterations in protein properties, structure and specific activity have been reported for fusion proteins relative to the native form [64,65,66]. In our case, both oxidoreductase activity and STAT-3 phosphorylase induction were impaired, suggesting that unfavorable interactions during folding of the fusion protein were responsible for this misfolding, as described previously [66]. To alleviate such structural disturbance, a GPGP linker has been inserted between Trx m and CT1 proteins [34,67]. However, in the present work, such a linker was not sufficient to separate both proteins effectively. Thus, linkers with different lengths or different conformations could be assessed in future works [66]. Alternatively, the introduction of a recognition sequence for a chemical agent or a protease between the fusion partner and the target protein would allow for site-specific cleavage of the fusion protein to remove the affinity fusion partner [68,69]. Generally, the fusion protein would be refolded properly and would recover its bioactivity when the tag was cleaved off [64]. However, this always introduces additional challenges into the downstream processing.

## 4. Materials and Methods 

### 4.1. Generation of Plastid Transformation Vectors

For the fusion strategy, *Nicotiana tabacum Trxm* lacking the stop codon and containing a His tag sequence at the N-terminus, was amplified by PCR as previously described [33]. PCR products were cloned into the pGEM-T vector (Promega Madison, WI, US). Likewise, the *ct1* sequence was cloned with *Sma*I and *Hind*III sites at the 5′ and 3′ ends, respectively, by PCR with the following primers: CT1-For 5′-CCCGGGATGAGCCGGAGGGAGGGAAG-3′ and CT1-Rev 5′-AAGCTTTTAGGCCGAGCCCCCGG-3′. *Trxm* and *ct1* sequences were fused together and to the promoter and 5′UTR of the tobacco *psbA* gene in a pKS intermediate vector (Promega). Finally, the fusion expression cassette was digested by *EcoR*I and *Hind*III and introduced into the pL3 vector [14]. The resulting vector was named pL3-Trxm-CT1.

For the co-expression strategy, the P*rrnG10L* promoter and leader region [14] were fused to the *Trxm* sequence at the 5′ end and cloned into the pGEM-T vector. The PrrnG10L-Trxm product was excised with *Not*I and introduced into the pL3-psbA-CT1 vector [14] upstream of the *ct1* gene. The resulting vector was named pL3-Trxm/CT1.

### 4.2. Bombardment and Regeneration of Chloroplast Transgenic Plants

Gold microprojectiles coated with the transformation vectors pL3-Trxm-CT1 and pL3-Trxm/CT1 were bombarded into in vitro-grown *N. tabacum* var. Petite Havana SR1 leaves (National Germplasm Resources Laboratory, Beltsville, MD, USA) using a PDS1000/He (Bio-Rad, Hercules, CA, USA) biolistic device, as described previously [37]. After bombardment, the leaves were incubated in the dark for 2 days at 28 °C, then cut into small pieces (around 5 × 5 mm) and placed adaxial side up on regeneration medium (RMOP) in Magenta vessels (Sigma, St Louis, MO, USA) containing 500 mg/L spectinomycin dihydrochloride (Duchefa Biochemie, Haarlem, Netherlands) as the selecting agent. The growth conditions of the culture chamber were 28 °C, 120 μmol m^−2^ s^−1^ and a 16-h photoperiod. Spectinomycin-resistant shoots were cut into small pieces (around 2 × 2 mm) and subjected to successive rounds of regeneration in the same selection medium. Finally, resistant shoots were rooted on growth medium containing 500 mg/L of spectinomycin and transferred into soil for homoplasmy confirmation and seed production.

Transformed and WT seeds were germinated on growth medium supplemented with or without 500 mg/L of spectinomycin, respectively. After 4–6 weeks, the seedlings were transferred to pots containing soil and grown in a greenhouse under natural sunlight.

### 4.3. Southern and Northern-Blot Analysis

Total plant DNA was extracted from young leaves of transformed and WT plants using the cetyltrimethylammonium bromide (CTAB) procedure [70]. Twenty micrograms of total DNA were digested with *Bgl*II, separated on a 0.8% (*w/v*) agarose gel and transferred to a nylon membrane. The membrane was hybridized with a 0.8 kbp probe (FS) homologous to the flanking sequences and specific probes against CT1 and Trx m sequences obtained as previously described [14,33]. Probe labeling and hybridization were performed using the DIG High Prime DNA Labeling and Detection Starter Kit II (Roche Applied Science, Mannheim, Germany). Bound probes were visualized using the chemiluminescent substrate CSDP provided in the kit. 

Total RNA was extracted from young leaves of transformed and WT plants using an Ultraspec RNA isolation kit (Biotecx Laboratories, Houston, TX, USA) according to the manufacturer’s instructions. Twenty micrograms of RNA were electrophoresed on 1.5% agarose/formaldehyde gels and transferred to a nylon membrane. The blot was hybridized with CT1- and Trx m-specific probes. Labeling and hybridization were performed using the chemiluminescent detection system mentioned above. Ethidium bromide-stained total leaf RNA was used to assess loading.

### 4.4. Western-Blot Analysis

Leaf discs from transformed and WT plants (around 200 mg) were finely powdered in liquid nitrogen, homogenized in Laemmli buffer (0.5 M Tris–HCl pH 6.5, 4% SDS, 20% glycerol and 10% β-mercaptoethanol) and boiled at 99 °C for 5 min. After 3 min of centrifugation at 14,000 rpm, the supernatant was considered the total protein (TP). The TP was quantified using the RC-DC protein assay (Bio-Rad, Hercules, CA, USA) with BSA as a standard, according to the manufacturer’s instructions. Leaf extracts were electrophoresed in a 13% polyacrylamide gel, and separated proteins were transferred to a nitrocellulose membrane for immunoblotting. Commercial human CT1 protein (PeproTech, London, UK) was used as positive control. Western blot analyses were performed using specific antibodies for CT1 (R&D Systems, Minneapolis, MN, USA) and Trx m [33]. Antibody dilution for anti-CT1 was 1:4000, and as a secondary antibody a peroxidase-conjugated anti-mouse antibody (Sigma-Aldrich) was used at 1:15,000 dilution. For anti-Trx m, dilutions were 1:5000 for primary antibody and 1:10,000 for secondary antibody (peroxidase-conjugated anti-rabbit antibody, Sigma-Aldrich). Detection was performed using an ECL Prime Western blotting system (GE Healthcare, Buckinghamshire, UK).

Quantification of recombinant CT1 was performed by comparing dilution series of TP extracted from young fully expanded leaves of transgenic plants with a dilution series of commercial human CT1 (PeproTech). For each protein, adequate amounts were loaded on an SDS–PAGE gel, electrophoretically separated and then analyzed by Western blot. Immunoblots were quantified using Gene-Tools analyzer software (SynGene, Cambridge, UK).

### 4.5. Bioactivity Analysis of Recombinant CT1

The human hepatoma cell line HepG2 was maintained in Dulbecco’s modified Eagle’s medium (Gibco, Paisley, UK) containing 10% fetal calf serum. For cell signaling experiments, cells were added to 6-well plates with 7.5 × 10^5^ cells/well. After adhesion, cells were cultured in serum-free medium for 48 h and then stimulated for 30 min with 25 ng of commercial human CT1 (PeproTech) or CT1 from crude extracts of transgenic plants. Cells treated with WT plant extracts and non-stimulated cells were used as negative controls. HepG2 cells were lysed for Western blot analysis in cold lysis buffer (50 mM Tris-HCl, pH 7.4, 150 mM KCl, 1% Nonidet P-40, 0.5% sodium deoxycholate, 0.1% SDS, 1 mM sodium orthovanadate, 10 mM sodium fluoride and a cocktail of anti-proteases). Protein from lysates was heat denatured in double-strength SDS sample buffer containing DTT before separation by SDS-PAGE. Western blot analysis was performed using specific antibodies for phosphorylated STAT-3 (Tyr705) (Cell Signaling Technology, Beverly, MA, USA) and actin (Sigma). Densitometric analysis was performed by chemiluminescence detection using an ImageQuant ECL system and ImageQuant TL software (GE Healthcare). The expression levels are presented as the fold increase relative to free-CT1 (= 1) using the ratios between the densities of the P-STAT-3 bands and the corresponding actin bands.

### 4.6. Trx m Protein Purification

Overexpressed His-tagged Trx m proteins were extracted from Trxm-CT1 or Trxm/CT1 tobacco leaf extracts in five volumes of extraction buffer (15.5 mM Na_2_HPO_4_, 4.5 mM NaH_2_PO_4_, 20mM NaCl, 25mM imidazole, 0.1% triton, and protease inhibitor cocktail tablets (Roche)) and purified by chromatography on affinity columns packed with Ni-NTA agarose (Qiagen, Hilden, Germany). The column was washed twice with a wash buffer (15.5 mM Na_2_HPO_4_, 4.5 mM NaH_2_PO_4_, 20 mM NaCl, and 45 mM imidazole). Elution was performed with the same buffer used for washing with the exception of the imidazole concentration, which was increased to 500 mM. Finally, Vivaspin 4 ultrafiltration spin columns (Sartorius Stedim Biotech, Goettingen, Germany) with a 10 kDa MWCO were used to dialyse the eluted protein against PBS buffer to avoid imidazole interactions in the subsequent assays. The protein concentration was measured using the RC-DC protein assay (Bio-Rad, Hercules, CA, USA), according to the manufacturer’s instructions.

### 4.7. Insulin Reduction Assay

The disulfide oxidoreductase activity of the His-tagged Trxm-CT1 or Trxm/CT1 proteins was determined according to the DTT-dependent insulin reduction assay [38]. Reactions were assayed at 25 °C and initiated by adding 0.5 mM DTT to the reaction mixture, which contained 100 mM Tris-HCl (pH 7.5), 2 mM EDTA, 1 mg/mL insulin (Sigma-Aldrich, St. Louis, MO, USA), and 4 µM of purified Trxm-CT1 or Trxm/CT1 proteins. Trx from *E. coli* (Sigma-Aldrich) was used at 1 and 3 µM as positive control. An assay in the absence of Trx was used as negative control. The oxidoreductase activity was determined by monitoring the increase in turbidity at 650 nm using a microplate reader (Multiskan Ex, Labsystems, Helsinki, Finland).
